# Resveratrol Attenuates Diquat-Induced Oxidative Stress by Regulating Gut Microbiota and Metabolome Characteristics in Piglets

**DOI:** 10.3389/fmicb.2021.695155

**Published:** 2021-07-12

**Authors:** Qingyao Fu, Zhen Tan, Liguang Shi, Wenjuan Xun

**Affiliations:** ^1^Hainan Key Lab of Tropical Animal Reproduction and Breeding and Epidemic Disease Research, College of Animal Science and Technology, Hainan University, Haikou, China; ^2^Tropical Crops Genetic Resources Institute, Chinese Academy of Tropical Agricultural Sciences, Danzhou, China

**Keywords:** metabolome, microbiota, oxidative stress, piglet, resveratrol

## Abstract

Previous studies have shown that dietary resveratrol (RES) reduces diarrhea and attenuates oxidative stress in piglets challenged with diquat. However, the effect of dietary resveratrol on the gut microbiota of these piglets, as well as the potential relationships between intestinal microflora and metabolites, remain unclear. Here, *16S* ribosomal DNA sequencing and metabolome analyses were performed to investigate the effect of RES on the gut microbiota and metabolome of diquat-challenged piglets. A total of 18 weaned piglets (aged 28 ± 2 days) were divided into the control group (basal diet), diquat group (basal diet + diquat challenge), and RES group (basal diet containing 90 mg/kg RES + diquat challenge). Compared with the control group, piglets in the diquat group showed enriched relative abundance of the phyla *Firmicutes* and *Actinobacteria*, the genus *Ruminococcaceae* UCG-005, and members of the *Eubacterium coprostanoligenes* group. Noteworthy, RES supplementation significantly reduced the levels of these microorganisms. In contrast, the relative abundance of some beneficial bacterial species in the RES group, such as the genera *Clostridium sensu stricto 1* and *Lachnospiraceae unclassified* were significantly higher than in the diquat and control groups. Metabolomic analysis indicated that some metabolites, including indole-3-carbinol, 5-hydroxyindole-3-acetic acid, and uridine, were significantly upregulated upon RES supplementation. In particular, the relative abundance of uridine, indole, and alpha- and beta-dihydroresveratrol was significantly higher in the RES group than in the control group. Moreover, most gut bacterial genera were found to be highly correlated with altered gut microbiota-related metabolites. These findings suggest that dietary supplementation with resveratrol may alter the composition and metabolites of colonic microbiota in diquat-challenged piglets, which provides important insights into the use of resveratrol as a feed additive for gut microbial regulation in piglets with inflammatory and oxidative stress-associated disorders.

## Introduction

Oxidative stress, which is caused by an imbalance between pro-oxidative and antioxidative factors, can decrease the antioxidant capacity of piglets and reduce the absorption of nutrients (Yin et al., 2014). Studies have found that the redox balance before and after weaning is disrupted in piglets, with antioxidant enzyme activity being significantly reduced, whereas free radicals and peroxidation products are significantly increased, indicating that weaning can induce oxidative stress (Cao et al., 2019; Zhang et al., 2020). Weaning also causes changes in the composition of the intestinal microbial community (Zhu et al., 2012; Qin et al., 2019). Oxidative stress caused by weaning could lead to gut bacterial dysbiosis, which increases the probability of diarrhea and intestinal infection in piglets, and reduces immunity and growth performance (Gresse et al., 2017; Tang et al., 2020). Furthermore, gut dysbiosis not only causes inflammation and oxidative stress but also reduces microbial metabolites butyrate and acetate (Yoo et al., 2020). There is evidence that many of metabolites are produced by the gut bacteria could inhibit the growth of pathogenic bacteria (Man et al., 2020b). The gut microbiota is known to play an evolutionarily conserved role in metabolism, immunity, development, and behavior of the host and has the ability to provide nutrition, optimize the intestinal immune system, regulate the growth of the intestinal epithelial mucosa, and protect the intestinal epithelial barrier (Wampach et al., 2017; Feng et al., 2018; Valdes et al., 2018). The intestinal microflora is dominated by diverse anaerobes, providing both a health benefit to the host and a barrier to infection (Eckburg et al., 2005). Therefore, feed additives that may relieve oxidative stress in piglets have gathered increased attention in the feed industry.

Antibiotics have been widely used in pig feed, especially in weaned piglets to promote growth, treat infectious diseases, and control the gut microbiota (Hu et al., 2020; Zeng et al., 2021). However, antibiotics not only affect the target microorganisms but also the microbial community, especially the gut microbiota. Studies have found that antibiotics can severely destroy the intestinal microbial community, including composition and function, and reduce the concentration of short-chain fatty acids in piglets (Yu et al., 2018; Hu et al., 2020). Antibiotics can also cause intestinal tight junction barrier dysfunction and activate the intestinal NOD-like receptor (NLR) family pyrin domain containing three mediated inflammasomes and autophagy in mice (Feng et al., 2019). In recent years, several studies have explored the relationship between polyphenolic compounds and gut microbes as a new research perspective.

Resveratrol (RES) is a plant polyphenol that belongs to the stilbene family of plant phytoalexin (Zhang et al., 2017; Wang et al., 2020). Several studies reported that resveratrol exerts strong biological activities, including antioxidative, anti-inflammatory, and antibacterial activities by regulating the composition and content of the intestinal flora (Breuss et al., 2019; Man et al., 2020a). Recently, RES was shown to alleviate intestinal inflammation by improving the proliferation of the beneficial bacteria *Lactobacillus* and *Bifidobacterium* in weaning piglets (Gan et al., 2019). Moreover, Li et al. (2020) found that resveratrol could inhibit the abnormal proliferation of Akkermansia and *Sutterella* and increase the relative abundance of *Bifidobacterium*, thus regulating intestinal microbes to reduce colitis-related inflammation in mice. Several studies have examined the influence of RES on the composition of the intestinal microbiome by addressing its lipid-lowering and anti-inflammatory effects (Qiao et al., 2014; Meng et al., 2019; Sun et al., 2019). This study aims to investigate whether resveratrol can alleviate oxidative stress in piglets by regulating their intestinal microflora and metabolites.

In this study, we used weaned piglets to establish an oxidative stress model. Subsequently, 16S rDNA sequencing and untargeted metabolomic correlation analysis were used to study the changes in the intestinal microbial community in piglets. Our research elucidates the regulatory mechanism of resveratrol in protecting the intestinal barrier against oxidative stress in piglets.

## Materials and Methods

### Experimental Design and Sample Collection

The study was approved by the Animal Care and Use Committee of Hainan University (HNUAUCC-2021-00003, Haikou, China). A total of 18 piglets (Duroc × Landrace × Yorkshire, 7.25 ± 0.13 kg) weaned at 28 days were randomly assigned to three treatments with six replicate pens/treatment: Basal diet (control), basal diet + diquat (diquat), and 90 mg/kg RES diet + diquat (RES). The diets were formulated to meet the nutrient requirement recommendations of the National Research Council (NRC, 2012) (Table 1). The piglets were individually housed in a nursery room with free access to feed and water. On day 15 after the initiation of treatment, piglets in the dietary treatment group were injected intraperitoneally with either 10 mg/kg body weight of diquat or the same volume of sterilized saline. On day 22, all piglets were killed by injection of sodium pentobarbital solution (50 mg/kg bodyweight). Colonic contents (middle section) were collected in 10 ml sterile centrifuge tubes and stored immediately at −80°C for subsequent analysis of microbiota composition and metabolites. RES was provided by Shanghai Yuanye Co., Ltd. (purity of 98%, Shanghai, China) and thoroughly mixed with the basal diet. Diquat was purchased from Sigma-Aldrich (Purity ≥ 99%, St. Louis, MO, United States).

**TABLE 1:** Composition and nutrient levels of the basal diet (air-dry basis) (%).

**Ingredients**	**Content**	**Nutrient levels**	**Content**
Corn	62.50	Digestible energy (MJ/kg)^b^	14.21
Soybean meal	20.50	CP (%)	19.62
Whey powder	5.50	Ca (%)	0.75
Fish meal	4.50	Available P (%)	0.40
Soybean oil	1.30	Lys (%)	1.31
Sucrose	1.50	Met (%)	0.42
Limestone	0.60	Thr (%)	0.73
Choline chloride	0.10	Trp (%)	0.20
CaHPO_4_	1.20		
Complex acidifying agent	0.30		
L-Lysine; HCl	0.40		
DL-methionine	0.16		
L-Tryptophan	0.04		
L-Threonine	0.10		
NaCl	0.30		
Vitamin–mineral premix^a^	1.00		
Total	100.00		

*^a^Provided per kilogram of diet: VA 2,700 IU, VD 3,300 IU, VE 25 IU, VK_3_ 0.5 mg, VB_1_ 10 mg, VB_2_ 20 mg, VB_12_ 80 mg, folic acid 0.3 mg, nicotinic 22 mg, pantothenate14 mg, biotin 0.05 mg, chorine 830 mg, Cu 5.6 mg, Fe 90 mg, Mn 5.2 mg, Zn 106 mg, Se 0.3 mg, I 0.2 mg.*

*^*b*^Digestible energy was calculated from data provide by Feed Database in China (2012), while the other nutrient levels were measured values.*

### DNA Extractions, Polymerase Chain Reaction Amplification, and 16S rDNA Sequencing

DNA from different samples was extracted using the E.Z.N.A. Stool DNA Kit (D4015, Omega, Biotek, Norcross, GA, United States) according to the manufacturer’s instructions. The total DNA was eluted in 50 μl of elution buffer and stored at −80°C until the polymerase chain reaction (PCR) were performed by LC-Bio Technology Co., Ltd., Hang Zhou. The 5′-ends of the primers were tagged with specific barcodes per sample and sequenced using universal primers. PCR amplification was performed in a total volume of 25 μl reaction mixture containing 25 ng of template DNA, 12.5 μl PCR premix, 2.5 μl of each primer, and PCR-grade water to adjust the volume. The PCR products were confirmed by 2% agarose gel electrophoresis, purified using AMPure XT beads (Beckman Coulter Genomics, Danvers, MA, United States), and quantified by Qubit (Invitrogen, Waltham, MA, United States). The amplicon pools were prepared for sequencing, and the size and quantity of the amplicon library were assessed on an Agilent 2100 Bioanalyzer (Agilent, Santa Clara, United States) and with the Library Quantification Kit for Illumina (Kapa Biosystems, Wilmington, MA, United States), respectively. The libraries were sequenced using the NovaSeq PE250 platform (Illumina) according to the manufacturer’s recommendations, provided by LC-Bio Technology.

### Metabolite Extraction and Untargeted Liquid Chromatography–Mass Spectrometry (LC-MS) Analysis

The collected samples were thawed on ice, and the metabolites were extracted using 50% methanol buffer. Liquid chromatography–mass spectrometry (LC-MS) analysis was performed on an ultra-performance LC system (SCIEX, Framingham, MA, United States) paired with a high-resolution tandem mass spectrometer TripleTOF5600plus (SCIEX). The quadrupole time-of-flight MS system was operated in both positive and negative ion modes. The detailed extraction and LC-MS analysis procedures were performed as reported by Wang et al. (2019).

LC-MS raw data files were converted into mzXML format and then processed using the XCMS-CAMERA workflow and metaX toolbox implemented with the R software (v 3.5.2)^1^. The online databases Kyoto Encyclopedia of Genes and Genomes (KEGG) and Human Metabolome Database (HMDB) were used to annotate the metabolites by matching the exact molecular mass data (*m*/*z*) of the samples with those from the database.

### Statistical Analysis

Alpha diversity and beta diversity were calculated by randomly normalizing to the same sequences. Then, according to the SILVA (release 132)^2^ classifier, the feature abundance was normalized using the relative abundance of each sample. Alpha diversity was applied to analyze the complexity of species diversity for a sample through five indices, including Chao1, observed species, Good’s coverage, Shannon, and Simpson, as determined by QIIME2^3^. Beta diversity was calculated using QIIME2, and graphs were drawn using the R package (v 3.5.2). BLAST was used for sequence alignment, and the feature sequences were annotated according to the SILVA database for each representative sequence. Other diagrams were implemented using the R package (v 3.5.2).

For the untargeted metabolomics, partial least-squares discriminant analysis (PLS-DA) and Spearman’s correlation analysis were used to explore differentially expressed metabolites that were screened and the significantly different genera obtained by *16S* rDNA sequencing analysis. Student’s *t*-test was used to calculate significant differences in metabolite concentrations between the two groups. Multiple test corrections were implemented by adjusting the *p*-values using a false-discovery rate (Benjamini–Hochberg) method. Supervised PLS-DA was conducted using metaX to discriminate among the different variables between groups, and the variable importance in projection (VIP) value was calculated. A VIP cutoff value of 1.0 was used to select important features (VIP ≥ 1; ratio ≥ 2 or ratio ≤ 1/2; *q*-value ≤ 0.05).

## Results

### Effect of RES on Diversity of Colonic Microbiota

After quality checks, demultiplexing, and assembly, a total of 1, 210, 428 valid sequences were obtained from each sample with a cutoff of 97% similarity using QIIME. Among the high-quality sequences, > 99% were 400–500 bp in length. Based on the efficient number of valid sequence numbers, the impact of diquat exposure and the regulatory roles of RES on dominant bacterial communities were investigated.

**FIGURE 1:**
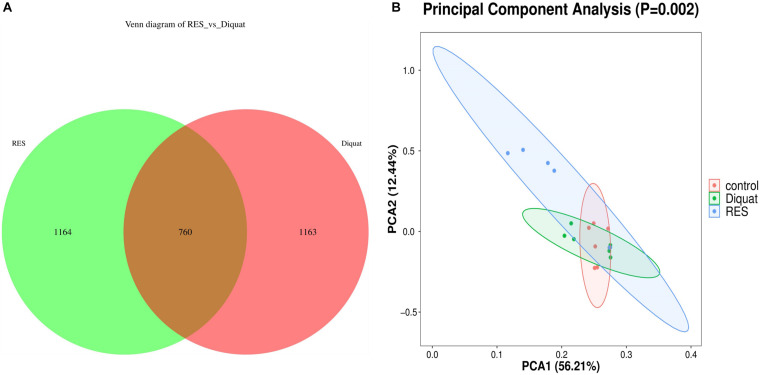
Gut microbiome diversity analysis. **(A)** Venn diagram shows the unique and shared OTUs in the RES and diquat groups (*n* = 6). **(B)** Principal component analysis (PCA) of bacterial community structures in different groups; each represented by one color (*n* = 6). RES, colonic microbiota of the supplementation resveratrol group; diquat, colonic microbiota of the diquat-challenged group. The same as fellow.

Colon samples of piglets from the diquat and RES groups showed 1,924 and 1,923 operational taxonomic units (OTUs), respectively. Among them, 760 shared common bacteria and 2,327 individual bacteria were isolated from the two groups (Figure 1A). Alpha diversity was calculated to evaluate the microbial community richness and diversity. However, as indicated by Good’s coverages, Chao1 index, observed species, Shannon index, and Simpson index, no significant differences were observed among the groups (Supplementary Figure 1). Regarding beta diversity, depicted as a principal component analysis (PCA) plot with PCA1 and PCA2 representing diet-induced variations and intragroup variations in microbial structure, respectively, the diquat-treated group showed similar diversity compared with the control group. However, the RES group clustered further away from the other experimental groups, indicating greater variation in beta diversity (Figure 1B).

### Effect of RES on Colonic Microbiota Composition and Abundance

**FIGURE 2:**
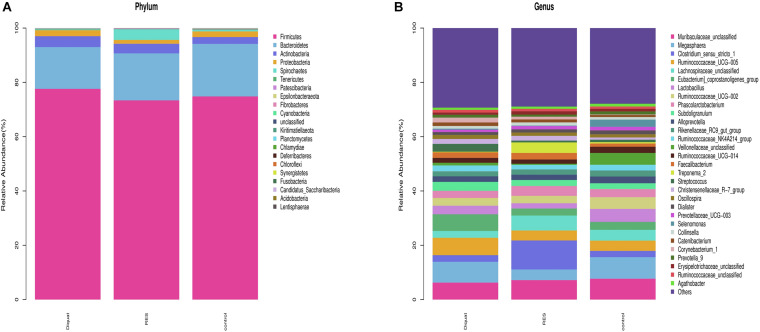
Gut microbiome composition and abundance analysis. **(A,B)** Histogram of the top 30 at phyla **(A)** and genera **(B)** in each group.

Taxonomic analysis showed that *Firmicutes*, *Bacteroidetes*, *Actinobacteria*, *Proteobacteria*, and *Spirochaetes* were the dominant phyla, accounting for > 99% of the total colonic bacteria. Of these predominant phyla, the abundance of *Firmicutes* (77.62 vs. 74.82%) and *Actinobacteria* (4.01 vs. 2.51%) was significantly increased, whereas the abundance of *Bacteroidetes* (15.40 vs. 19.35%) was significantly decreased in the diquat-treated animals compared with the control group. However, the RES group tended to have increased abundance of *Bacteroidetes* (17.29%) and decreased abundance of *Firmicutes* (73.38%) and *Actinobacteria* (3.51%) compared with the diquat group. In addition, the RES group has higher abundance of *Spirochaetes* and lower *Proteobacteria* than the control and diquat groups (Figure 2A).

At the genus level, the relative microfloral abundance differed among the groups (Figure 2B). Compared with the control group, the abundance of *Ruminococcaceae* UCG-005 (6.42 vs. 3.79%) and *Eubacterium coprostanoligenes* (6.15 vs. 2.92%) were significantly increased in the diquat group. In contrast, RES supplementation decreased the abundance of *Ruminococcaceae* UCG-005 (3.69 vs. 3.79%) and *Eubacterium coprostanoligenes* (2.59 vs. 2.92%) compared with the diquat group. Furthermore, the abundance of *Megasphaera* (3.91 vs. 7.93%) was significantly lower in the RES group than in the control group. The relative abundance of *Clostridium_sensu_stricto_1* (10.69, 2.44, and 2.32%, respectively), *Lachnospiraceae_unclassified* (5.49, 2.49, and 3.97%, respectively), and *Treponema_2* (3.88, 0.21, and 0.50%, respectively) in the RES group was significantly higher than in the diquat and control group.

### Effect of RES on Colonic Microbiota Function

**FIGURE 3:**
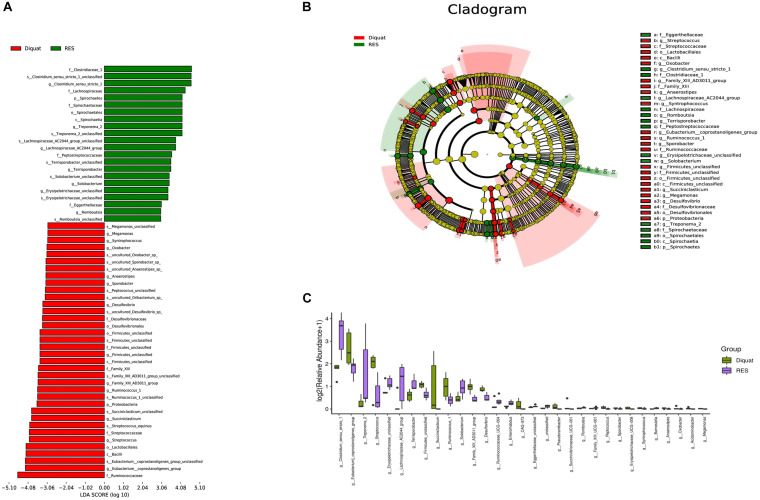
Gut microbiome significantly different microbe analysis. **(A,B)** Significantly different microbes **(A)** and the cladogram **(B)** by LEfSe analysis. **(C)** Barplot shows microorganisms with significant differences at genus level between diquat and RES groups.

Diquat challenge significantly increased the abundance of *Eubacterium coprostanoligenes* and *Streptococcus* compared with the RES group (Figure 3A). In contrast, compared with the diquat group, *Clostridium sensu stricto* (cluster 1), *Erysipelotrichaceae* UCG-009, and *Lachnospiraceae* AC2044 groups were significantly upregulated in the RES group. Next, linear discriminant analysis effect size (LEfSe) was used to identify statistically significant biomarkers among the different groups. In the colon, 22 and 36 different taxonomic levels of microorganisms were identified as potential biomarkers between the RES and diquat groups, respectively (Figure 3B). In the microbial cladogram, the significantly different microorganisms were mainly classified as belonging to the phylum *Proteobacteria* in the diquat group and the phylum *Spirochaetes* in the RES group (Figure 3C).

**FIGURE 4:**
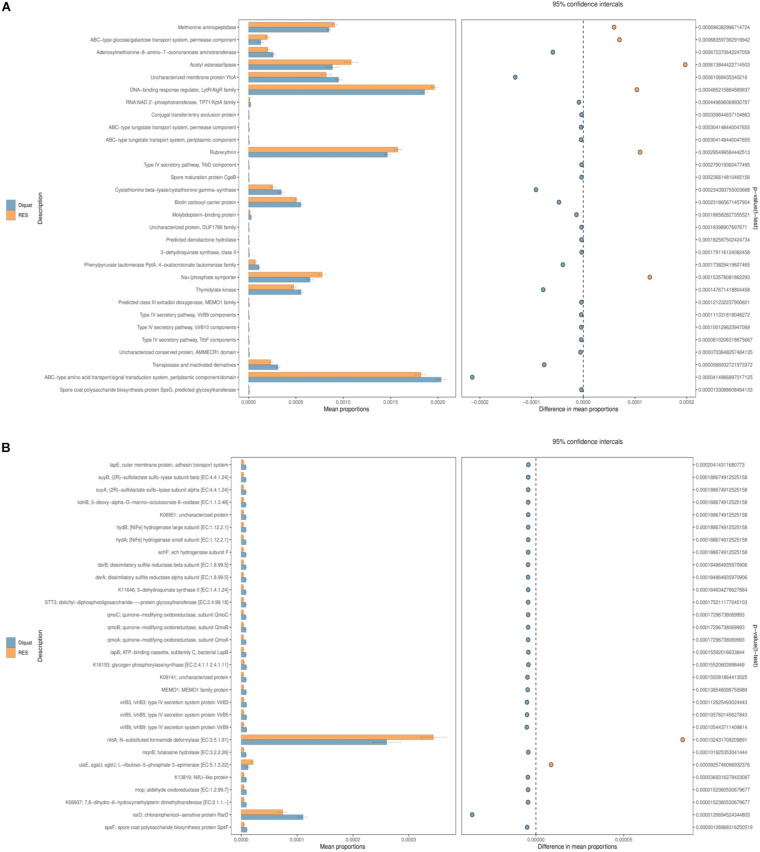
Gut microbiome comparison of PICRUSt-predicted functional pathway analysis. **(A,B)** Phylogenetic Investigation of Communities by Reconstruction of Unobserved States (PICRUSt2), which will be used to analysis of the difference of COG **(A)** and KO **(B)** metabolic pathway between diquat and RES groups at the second level.

A total of 30 second-level classification Clusters of Orthologous Genes (COG) and KEGG Orthology (KO) pathways were verified based on the structure of the colon microbiota established using the PICRUSt2 algorithm. “Methionine aminopeptidase,” “ABC-type amino acid transport/signal transduction system, periplasmic component/domain,” and “adenosylmethionine-8-amino-7-oxononanoate aminotrans- ferase” were the top 3 functional annotations in both diquat and RES groups. The abundance of six functional annotations in the RES group was higher than that in the diquat group, including “methionine aminopeptidase,” “ABC-type amino acid transport/signal transduction system, periplasmic component/domain,” “DNA-binding response regulator, LytR/AlgR family,” “acetyl esterase/lipase,” “rubrerythrin,” and “Na^+^/phosphate symporter.” The abundance of the other 24 functional annotations was higher in the diquat group (Figure 4A). For the KO pathways, only two functional annotations were higher in the RES group than in the diquat group, which were the “nfdA; N-substituted formamide deformylase [EC:3.5.1.91]” and “rarD; chloramphenicol-sensitive protein RarD” (Figure 4B).

### Effect of RES on Colonic Metabolites

**FIGURE 5:**
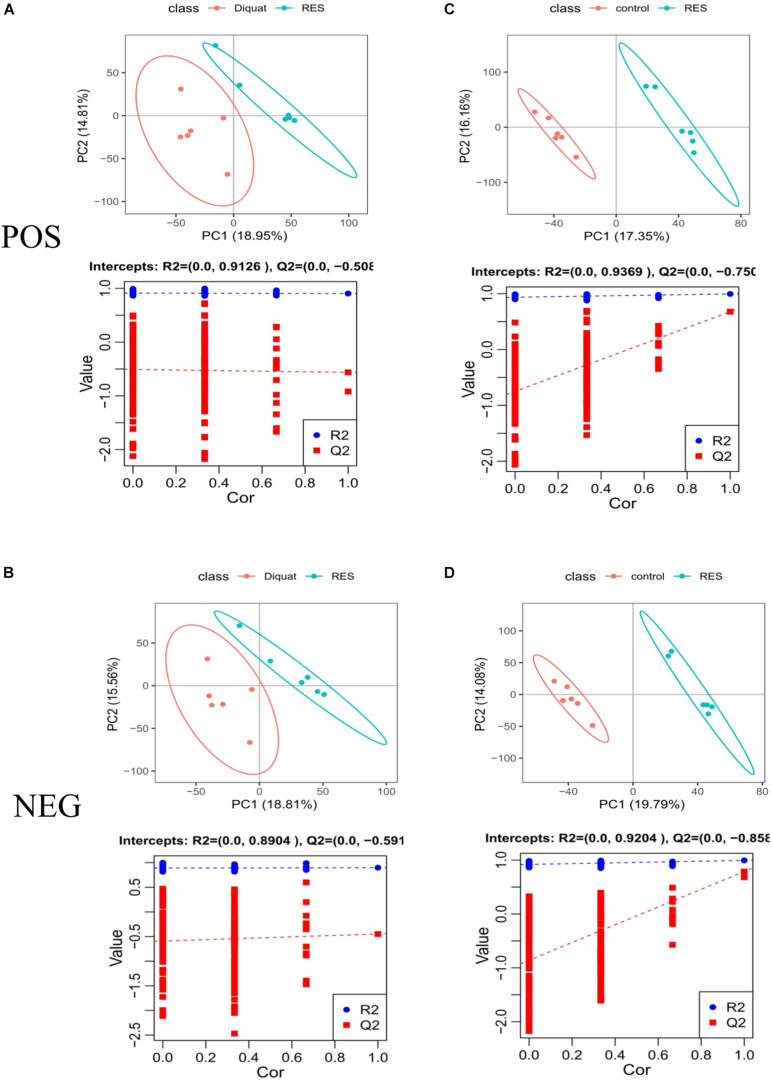
Positive model **(A,C)** and negative model **(B,D)**. PLS-DA scores: The abscissa represents the first principal component PC1, and the ordinate represents the second principal component PC2. Replacement test diagram: R2 stands for model verification, and the Y matrix of original classification and *N* times of different arrangement are linearly regressed with R2Y and Q2Y, and the intercept values of regression line and *y*-axis are R2 and Q2, respectively; used to measure whether the model is over-fitted.

To explore changes in metabolites induced by RES supplementation in diquat-challenged piglets, a metabolome program was carried out. All metabolites identified were assigned to the KEGG and HMDB databases. In the KEGG databases, 11,052 and 6,524 metabolites were classified into 21 KEGG second-grade pathways, 4,806 and 3,906 of which were classified into “metabolism” in the positive and negative ion models, respectively (Supplementary Table 1). To further explore the effect of dietary RES supplementation on metabolites in the colonic microbiota, PLS-DA was performed. This analysis revealed metabolic differences between RES and diquat groups (Figures 5A,B), as well as RES and control groups (Figures 5C,D), suggesting that RES leads to significant biochemical changes in the colon.

In total, 10 and 12 differentially enriched metabolites were identified between the RES and diquat groups based on VIP values in the positive and negative ion models, respectively. In the positive ion model, nine metabolites were upregulated and one metabolite was downregulated in the RES compared with the diquat group (Table 2), while eight metabolites were upregulated and four were downregulated in the negative ion model (Table 3). For example, indole-3-carbinol, 5-hydroxyindole-3-acetic acid, and uridine were higher in the RES group than in the diquat group. Similarly, there were 45 and 52 differently enriched metabolites between the RES and control groups in both models, of which 33 were upregulated and 12 were downregulated in the positive ion model and 36 were upregulated and 16 were downregulated in the negative ion model. Moreover, compared with the control group, the relative abundance of 5-hydroxyindole-3-acetic acid, uridine, indole, 5-hydroxyindole, and alpha- and beta-dihydroresveratrol was increased in the RES group (Supplementary Table 2).

**TABLE 2:** Statistics of identified metabolites: Differences in metabolites in the colon of RES and diquat groups in positive ion model.

**POS–MS2 metabolite**	**VIP**	***p*-value**	**Regulated**	**Ratio**
D-Pipecolinic acid	2.214461237	0.005198698	Up	2.5508088
D-Pipecolinic acid	2.074820236	0.010427436	Up	2.383560707
Quinaldic acid	2.317940294	0.034151319	Up	2.980914999
Kynurenic acid	2.944953358	0.022946148	Up	3.881648746
Kynurenic acid	2.564821943	0.043836902	Up	2.381451396
5-Hydroxyindole-3-acetic acid	2.065636737	0.015984145	Up	2.31297022
Thr-Pro	2.379122724	0.038659913	Up	2.789520415
Isoleptospermone	1.92749486	0.047965814	Up	2.136892568
Hecogenin	2.409969617	0.028175839	Up	2.708499837
Adenine	2.468348533	0.000251114	Down	0.43864468

*Color words represent significantly different metabolites.*

**TABLE 3:** Statistics of identified metabolites: Differences in metabolites in the colon of RES and diquat groups in negative ion model.

**NEG–MS2 metabolite**	**VIP**	***p*-value**	**Regulated**	**Ratio**
4-Hydroxyquinoline	3.343348598	0.00335143	Up	4.537624218
4-Hydroxyquinoline	3.277758768	0.007928362	Up	4.383843998
4-Hydroxyquinoline	2.404751452	0.004493916	Up	2.865032578
Indole-3-carbinol	2.257573036	0.013929149	Up	2.493885614
Kynurenic acid	4.25504284	0.012883298	Up	6.175671957
8-Hydroxyquinoline-5-carboxylic acid	3.213613744	0.011805355	Up	4.259385127
Uridine	2.212051715	0.029524354	Up	3.006658051
(9S,10S)-10-hydroxy-9-(phosphonooxy)octadecanoate	2.416704069	0.002106187	Up	2.285745048
2,4-Dimethylpimelic acid	1.901374613	0.047788102	Down	0.41094935
5-Hexyltetrahydro-2-furanoctanoic acid	2.124527012	0.011870781	Down	0.483194111
Pentadecanoylglycine	2.079969274	0.013569506	Down	0.487851469
3b,8a-Dihydroxy-6b-angeloyloxy-7(11)-eremophilen-12,8-olide	2.226676246	0.038921568	Down	0.253226068

*Color words represent significantly different metabolites.*

### Correlation Analysis Between Colonic Microbiota and Metabolites

**FIGURE 6:**
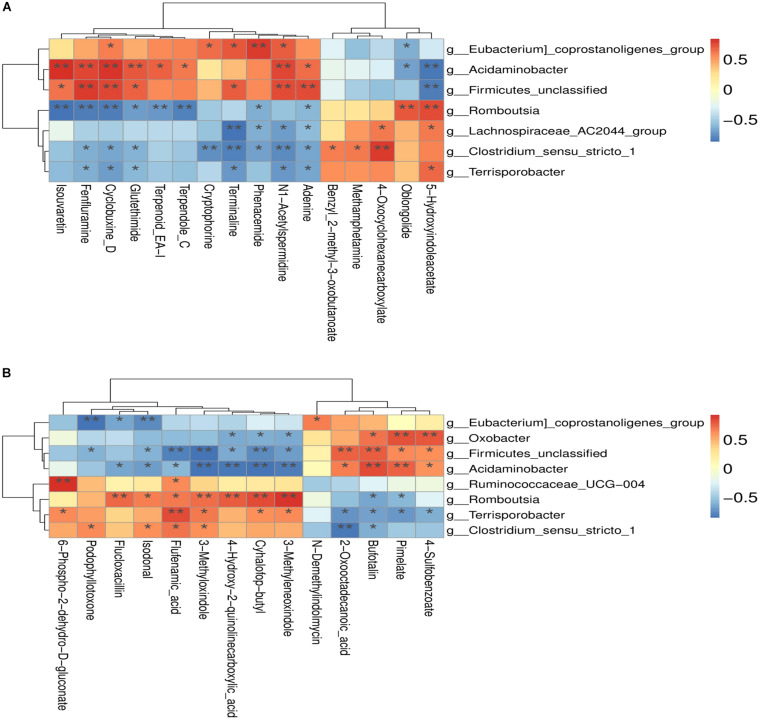
**(A)** Positive model; **(B)** negative model. **(A)** The correlation between positive ion metabolites and differential flora; **(B)** the correlation between negative ion metabolites and differential flora. *p-value < 0.05; **p-value < 0.01.

The correlation between the metabolites and the relative abundance of bacteria in the colon of both RES and diquat groups were assessed next. The levels of N1-acetylspermidine were positively correlated with *Firmicutes unclassified*, *Acidaminobacter*, and *Eubacterium coprostanoligenes* and negatively correlated with *Clostridium sensu stricto* 1 and *Lachnospiraceae* AC2044 group in the positive model (Figure 6A). In addition, it was observed that 3-methyloxindol and flufenamic acid were positively correlated with the *Clostridium sensu stricto* 1 but negatively correlated with *Firmicutes unclassified* and *Acidaminobacter* in the negative model (Figure 6B). Overall, these findings agreed with the observed taxa enrichment and the presence of metabolites in the colon.

## Discussion

Diet is a critical modulator of gut microbial composition and diversity, and it regulates oxidative stress and metabolism, thus exerting a beneficial effect on the host (Llewellyn et al., 2018). To evaluate the effect of RES on the intestinal microbiome, colonic microbiota were extracted for *16S* rDNA analysis. Microbial diversity is considered a new biomarker that reflects host health and stability (Clarke et al., 2014). In the present study, diquat and RES exposure did not affect the alpha diversity of the gut microbiome of piglets, suggesting that the ecological diversity of the colonic microbiota were similar, regardless of the treatments. These findings are consistent with those of Meng et al. (2019), who found that maternal dietary RES failed to modify the fecal microbiota alpha diversity in weaned piglets.

*Firmicutes*, *Bacteroidetes*, *Proteobacteria*, and *Actinobacteria* are the most predominant phyla in pigs and humans (Yatsunenko et al., 2012; Niu et al., 2015; Chen T. et al., 2019). In agreement with previous results, the current study also found that these bacteria were the most abundant intestinal microorganisms in piglets. Oxidative stress may not only induce the growth of pathogenic bacteria (such as *Proteobacteria* and *Campylobacter*) but also cause malabsorption of nutrients and inflammation (Merrifield et al., 2013; Frese et al., 2015; Xiong et al., 2019). In the present study, diquat-challenged piglets had a higher abundance of *Firmicutes* and *Actinobacteria* but lower abundance of *Bacteroidetes* than the control group. Supplementation with RES prevented the increase in *Firmicutes* and *Actinobacteria*, which suggests that diquat exposure changed the bacterial composition and distribution. It is widely recognized that most bacteria of the *phylum Proteobacteria* can cause sustained intestinal inflammation and injury in piglets (Chen J. et al., 2019). Given the RES-induced significant decrease in the abundance of *Proteobacteria* herein described, dietary RES supplementation maybe a useful strategy to alleviate intestinal injury by decreasing the proliferation of pathogenic bacteria.

At the genus level, the present results revealed high abundance of *Ruminococcaceae* UCG-005 and *Eubacterium coprostanoligenes* in the diquat group compared with the control group. A previous study reported that *Ruminococcaceae* UCG-005 are a stable microbiota component of the caecum and colon in piglets (Liu et al., 2018). Mateos et al. (2018) found that *Ruminococcaceae* UCG-005 are positively correlated with chronic inflammation, metabolic diseases, and mycotoxin exposure in weaned piglets. Moreover, Hung et al. (2019) found that *Ruminococcaceae* UCG-005 are positively correlated with the incidence of diarrhea. Therefore, the rise in *Ruminococcaceae* UCG-005 by diquat challenge in this study may reflect the deterioration of the intestinal environment in the piglets as the abundance of *Ruminococcus* genera in the gastrointestinal tract is a significant factor contributing to the incidence of diarrhea in weaned piglets (Gaukroger et al., 2020). This result is consistent with our previous research that diquat challenge increased fecal score and caused diarrhea in piglets (Xun et al., 2020). *Eubacterium coprostanoligenes* are well known for their ability to convert cholesterol to coprostanol and reduce serum cholesterol. He et al. (2019) found the abundance of *Eubacterium coprostanoligenes* increased with an increase in serum cholesterol levels in heat stress-challenged piglets. Herein, the abundance of *Eubacterium coprostanoligenes* at the genus level increased upon diquat exposure as compared with the control group. Thus, it is possible that the relative abundance of *Eubacterium coprostanoligenes* increases when piglets are under oxidative stress. The *Clostridium* genus was divided into two major groups: *Clostridium sensu stricto* (clusters 1 and 2). *Clostridium sensu stricto* 2 was more abundant in diarrheal piglets than in healthy piglets, whereas *Clostridium sensu stricto* 1 showed the opposite results (Han et al., 2019). Moreover, *Clostridium sensu stricto* 1 was reported to consume mucus-derived saccharides as energy sources to produce short-chain fatty acids and promote the intestinal mucus barrier against pathogen adherence (Wlodarska et al., 2015). In the present study, the abundance of *Clostridium sensu stricto* 1 and *Lachnospiraceae* AC2044 was significantly higher in the RES group than in the diquat group, further suggesting that RES could enhance the intestinal antioxidative capacity by ameliorating the microbiota composition in the colon of piglets.

The antioxidant defense enzyme, adenosylmethionine-8-amino-7-oxononanoate aminotransferase, belongs to the pyridoxal phosphate-dependent aspartate aminotransferase superfamily. It is also a key enzyme in the nitrogen metabolism of all organisms (Jensen and Gu, 1996; Kanani et al., 2016). In the present study, the top 3 functional annotations were methionine aminopeptidase, ABC-type amino acid transport/signal transduction system, periplasmic component/domain, and adenosylmethionine-8-amino-7-oxononanoate aminotransferase in the pathway analysis of the Clusters of Orthologous Genes. Moreover, the relative abundance of rubrerythrin functional annotation in the RES group was found to be higher than that in the diquat group. Lumppio et al. (2001) demonstrated that rubrerythrin was classified as an alternative superoxide dismutase to the oxidative stress protection system. Therefore, it is reasonable to hypothesize that the addition of RES could reduce oxidative stress in piglets by regulating the construction and functional response of the colonic microbiome.

In this study, the effect of dietary RES supplementation on the gut metabolic profiles was studied by LC-MS analysis. The collected data confirmed that indoles and their derivatives play an important role in preventing gastrointestinal stress-induced lesions, regulating the expression of proinflammatory genes, and enhancing the barrier function of epithelial cells (Yokoyama and Carlson, 1979; Lee and Lee, 2010; Zheng et al., 2011). The 3-methyldioxyindole is an oxidation product of 3-methylindole *in vivo.* It is produced by bacteria in the colon and involved in the tryptophan metabolism. Zhao et al. (2013) reported that 3-methyldioxyindole was significantly decreased in rat model of the chronic kidney disease. In our study, an obvious decrease in 3-methyldioxyindole was also observed in the diquat group. In contrast, RES supplementation upregulated indole, 5-hydroxyindole, indole-3-carbinol, 5-hydroxyindole-3-acetic acid, uridine, and alpha- and beta-dihydroresveratrol compared with the diquat and control groups. Indeed, as a special type of signaling molecule, indole can exert anti-inflammatory activities in the intestinal tract of the host. Sun et al. (2020) reported that controlled addition of indole to enteropathy in a murine model attenuated inflammation and restored the intestinal flora balance. Uridine, a gastrointestinal metabolite of uridine monophosphate *in vivo*, is derived from the degradation of RNA. A previous study showed that maternal uridine supplementation could improve the intestinal barrier integrity and modify the apoptosis levels in the intestine of weaned piglets (Gao et al., 2020), thus potentially relieving the oxidative stress and improving the intestinal health of piglets. Hence, RES supplementation may strengthen the intestinal barrier and gut homeostasis by regulating the gut metabolite profiles.

Correlation analysis facilitated the identification of several bacterial genera that are potentially implicated in host metabolism. In the positive model, *Acidaminobacter* abundance was negatively correlated with two metabolites and positively correlated with eight metabolites, whereas *Terrisporobacter* was positively and negatively correlated with one and six metabolites, respectively. Altogether, the disruption of gut microbial composition and metabolic homeostasis could be the major underlying factor inducing the decline in the antioxidant capacity of diquat-challenged piglets, whereas RES can protect the intestinal health of the piglets by regulating gut microbiota and metabolome characteristics.

## Conclusion

The present study demonstrates that oxidative stress can lead to increase abundance of pathogenic bacteria, alterations in the colonic microbiota, and metabolome profiles. Conversely, dietary RES supplementation may play a beneficial role in the intestinal health of piglets affected by oxidative stress *via* regulation of the composition and metabolite profiles of the intestinal microbiome. Future research should further explore the underlying mechanisms that drive the interaction between colon bacteria and metabolites.

## Data Availability Statement

The data presented in the study are deposited in the NCBI SRA BioProject repository, accession number PRJNA723726.

## Ethics Statement

The study was approved by the Animal Care and Use Committee of Hainan University (Haikou, China).

## Author Contributions

WX: conception and design. LS, WX, and QF: animal feeding, sampling, and determination. QF and ZT: data analysis. QF and WX: drafting the manuscript. QF, ZT, LS, and WX: final approval of the manuscript. All authors contributed to the article and approved the submitted version.

## Conflict of Interest

The authors declare that the research was conducted in the absence of any commercial or financial relationships that could be construed as a potential conflict of interest.

## Footnotes

1 https://www.R-project.org

2 https://www.arb-silva.de

3 https://qiime2.org

## Supplementary Material

Supplementary Figure 1 The boxplot of differences on bacterial community diversity and richness. **(A)** Good’s coverage index; **(B)** Chao1 index; **(C)** Observe species index; **(D)** Shannon index; **(E)** Simpson index. ^∗^*p* < 0.05, significant difference among different groups; none ^∗^ means no significant difference.

Supplementary Table 1 Statistics of identified metabolites.

Supplementary Table 2 Statistics of identified metabolites: Differences in metabolites in the colon of RES and control groups in positive ion **(A)** and negative ion model **(B)**, respectively.
