# Supplementary material for: Resveratrol Attenuates Diquat-Induced Oxidative Stress by Regulating Gut Microbiota and Metabolome Characteristics in Piglets
Source: Front Microbiol. 2021 Jul 12;12:695155. doi: 10.3389/fmicb.2021.695155 (PMC8312259; doi:10.3389/fmicb.2021.695155)
Supplement: Supplementary Figure 1 — The boxplot of differences on bacterial community diversity and richness. (A) Good’s coverage index; (B) Chao1 index; (C) Observe species index; (D) Shannon index; (E) Simpson index. ∗p < 0.05, significant difference among different groups; none ∗ means no significant difference. [file Data_Sheet_1.pdf]

Supplementary information:

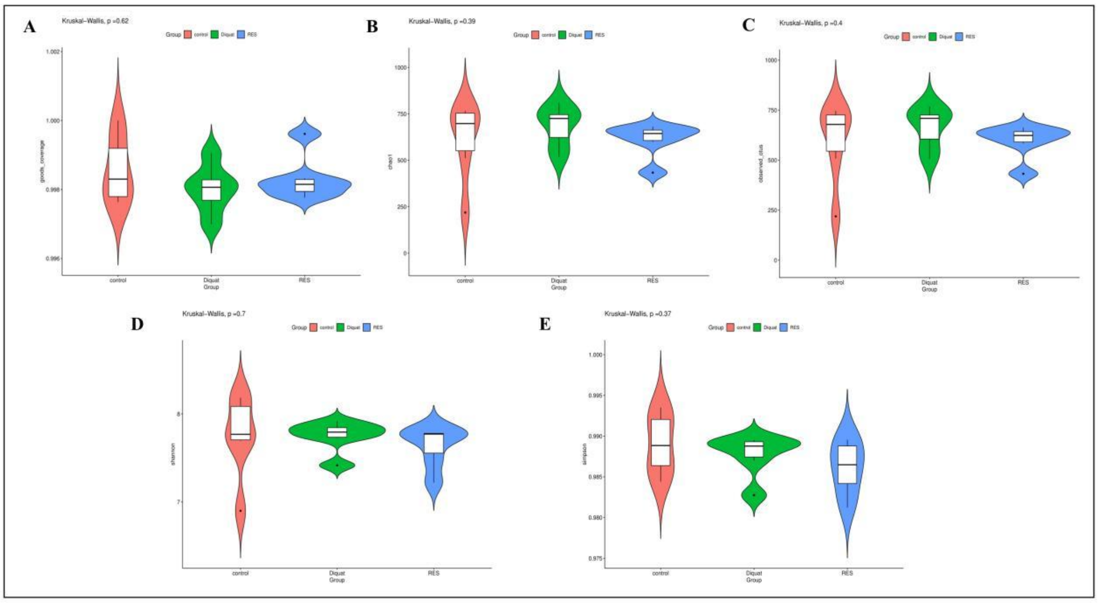

Supplementary Figure 1: The boxplot of differences on bacterial community diversity and richness. A.Good Coverages index; B.Chao1 index; C.Obeserve Species index; D.Shannon index; E.Simpson index. *Means significant difference (P< 0.05) among different groups, none * Means no significant difference.

Supplementary Table 1. Statistics of identified metabolites

Mode	All feature	All annotated	MS2	HMDB	KEGG
POS	19557	13946	1074	12466	11052
NEG	13552	8670	768	7447	6524

Supplementary Table 2. Statistics of identified metabolites: Differences in metabolites in the colon of RES and control groups in positive ion **(A)** and negetive ion model **(B)**, respectively.

POS--MS2 Metabolite **(A)**	VIP	*P*-value	Regulated	Ratio
Indole	2.515138987	0.136347769	up	2.636600633
1-(1-Pyrrolidinyl)-2-propanone	2.049794182	0.337636375	up	2.790697361
Isoquinoline	1.922405271	0.162317817	up	2.145815594
5-Hydroxyindole	1.756415842	0.347919252	up	2.214329193
2-Butyl-3-methylpyrazine	2.332095133	0.058806016	up	2.850727335
5-Aminopentanoic acid	1.857362511	0.365163407	up	3.034063772
5-Acetamidovalerate	2.065848629	0.264093995	up	2.22970033
2-Indolecarboxylic acid	2.101072896	0.38755169	up	3.378040118
Tryptophanol	1.908662851	0.136347769	up	2.128452066
4,5-Dihydro-1-benzoxepin-3(2H)-one	1.836235064	0.107728865	up	2.162562719
DL-.beta.-Homophenylalanine	2.285467832	0.244617189	up	5.160112743
Glycyl-L-norleucine	1.941472427	0.127158985	up	2.231378527
1-hydroxyhexanoylglycine	2.224239756	0.227641704	up	3.210751572
Kynurenic acid	3.0441392	0.135479455	up	7.381240572

Kynurenic acid	3.313537279	0.090082694	up	6.701602931
Atractylenolide III	2.579488881	0.312165735	up	3.092875213
Daidzein	2.019804919	0.162858416	up	2.387061339
Isoleptospermone	2.795293144	0.234890317	up	5.275104197
Met-His	2.805086492	0.022199065	up	3.762887256
Androstadienone	2.376750397	0.172340217	up	3.146357861
trans-Hydroxyperhexiline	4.087327387	0.00260258	up	12.80328772
Palmitoyl ethanolamide	2.141798238	0.235523138	up	2.466870361
Palmitoyl ethanolamide	2.157240064	0.311203531	up	2.665154024
11,14,17-Eicosatrienoic acid, (Z,Z,Z)-	2.091177516	0.356305714	up	4.081678928
8Z,14Z-Eicosadienoic acid	2.031919271	0.136347769	up	2.222958008
13-HDoHE	2.168647506	0.250009856	up	3.053217688
13-HDoHE	2.155803773	0.147948235	up	3.073229311
12-Ketodeoxycholic acid	1.841254011	0.182621121	up	2.01567866
Retinamide, N-(4-hydroxyphenyl)-	3.037506482	0.189372952	up	5.339494009
Soyasaponin Bb	1.930789614	0.322842216	up	2.615244916
Pilocarpine	2.97351591	0.143012437	up	5.887538897
Phenylalanyl-Glycine	3.432903141	0.163425374	up	6.202350224
alpha,beta-Dihydroresveratrol	1.623230015	0.368783257	up	2.320029751
Isonicotinic acid	1.543451438	0.353674458	down	0.498060133
Adenine	2.331933287	0.058806016	down	0.396017255
9-Azabicyclo[3.3.1]nonan-3-one	2.789999989	0.027076093	down	0.254212573
Tranexamic Acid	2.833735045	0.060258522	down	0.233483523
3-Methyldioxyindole	2.301590211	0.190932992	down	0.342671009
3-Methyldioxyindole	2.154451996	0.166235858	down	0.405037182
3-Methyldioxyindole	2.138986272	0.075750403	down	0.430224442
Triethyl phosphate	1.746680927	0.349027125	down	0.455570353
Ethyl 4-phenylbutanoate	1.852739764	0.097876238	down	0.494488076
Deoxyadenosine	2.22005164	0.068136102	down	0.405969233
3-Furancarboxylic acid, tetrahydro-4-methylene-2-octyl-5-oxo-, (2R,3S)-rel-	2.658775756	0.222202915	down	0.37843916
Testosterone decanoate	1.58111437	0.347618155	down	0.474820752

NEG--MS2 Metabolite **(B)**	VIP	*P*-value	Regulated	Ratio
Dimethylmalonic acid	1.925022212	0.178885707	up	2.580701953
Glutaric acid	2.451146682	0.08212352	up	4.047587587
4-Hydroxyquinoline	2.820219337	0.06574483	up	6.978537883
4-Hydroxyquinoline	3.166824267	0.073818855	up	8.734620655
N-Acetylvaline	1.745876872	0.199628651	up	2.144477629
N-Acetylvaline	1.972659261	0.194404598	up	2.368727556
4-Acetyl-2(3H)-5-benzoxazolone	1.672276912	0.274251368	up	2.463238098
Kynurenic acid	3.024930558	0.08559284	up	12.16821639

8-Hydroxyquinoline-5-9-carboxylic acid	3.215460191	0.08559284	up	9.336482646
5-Hydroxyindole-3-acetic acid	2.251042041	0.19797187	up	4.407572249
Xanthurenic acid	1.775982429	0.18793265	up	2.59930699
4-(2-Aminophenyl)-2,5-4-dioxobutanoic acid	2.531123573	0.259463329	up	6.205121614
Gly-Phe	2.289015856	0.058150153	up	3.313809859
4-Acetyl-2(3H)-benzoxazolone	2.283385326	0.316206507	up	7.038126965
Uridine	1.383799811	0.315804374	up	2.00599745
3-Hydroxydodecanedioic acid	2.015221951	0.040948116	up	2.313266972
Daidzein	1.888004287	0.217108947	up	3.296475573
6-Hydroxypentadecanedioic acid	1.533609172	0.267545635	up	2.328482691
Uridine	1.885601186	0.244965678	up	3.215158334
13-OxoODE	1.466409738	0.318274933	up	2.146987339
Alpha-dimorphecolic acid	2.834818198	0.062701777	up	6.81964333
Cis-8,11,14,17-Eicosatetraenoic acid	1.928855745	0.123888323	up	2.506581748
Verimol A	2.367119739	0.083628057	up	3.510700625
Corchorifatty acid F	1.977687389	0.286716174	up	4.929243243
Docosahexaenoic acid	1.946852931	0.091542933	up	2.661028037
Aflatoxin B1 dialcohol	3.057374056	0.1714814	up	5.661403685
Aflatoxin B1 dialcohol	2.360127935	0.223358592	up	3.210500647
2-Methyl-5-(8,11-pentadecadienyl)3--1,3-benzenediol	2.153136676	0.125328556	up	3.368550093
(9S,10S)-10-hydroxy-9-(phosphonooxy)octadecanoate	1.533764618	0.125142619	up	2.021235305
1b,3a,12a-Trihydroxy-5b-cholanoic acid	1.93722189	0.219909015	up	2.692677663
1b,3a,12a-Trihydroxy-5b-cholanoic acid	1.973164332	0.222288591	up	2.850457436
FAHFA 27:4; FAHFA(3:0/24:4)	2.202963513	0.261566263	up	4.082496308
1b,3a,12a-Trihydroxy-5b-cholanoic acid	2.338503669	0.212398086	up	3.314502688
13'-Carboxy-alpha-tocotrienol	2.460610506	0.169378125	up	5.391969309
3a,7b,12a-Trihydroxyoxocholanyl-Glycine	2.339376625	0.165912638	up	5.521754717
Echinocystic acid-3-O-glucoside	2.267264167	0.080315563	up	3.574283816
3-Methylpentanoic acid	3.884604269	0.016753825	down	0.048936932
xi-5-Acetyltetrahydro-2(3H)-furanone	1.630248616	0.152346565	down	0.498894144
D-(+)-Malic acid	1.6519845	0.258039768	down	0.471250323
2-Methylglutaric acid	1.650855285	0.176074647	down	0.467182877

3-Methyladipic acid	3.358119904	0.007653456	down	0.103292896
4-(3-Pyridyl)-3-<br>5-butenoic acid	2.150233768	0.227393063	down	0.324186922
3-Hydroxycinnamic acid	2.070731747	0.222356823	down	0.310403774
3-Dehydroquinic acid	1.566199508	0.21412228	down	0.497688941
3-Hydroxysebacic acid	1.930062863	0.217092786	down	0.327182354
2,4-Dimethylpimelic acid	1.742661305	0.08559284	down	0.413693112
Coriandrone C	1.91973274	0.240455375	down	0.412353029
Tetradecanedioic acid	1.773804402	0.263216346	down	0.492628459
Enterolactone	2.232922021	0.190290774	down	0.295790895
3b,8a-Dihydroxy-6b-angeloyloxy-7(11)-<br>eremophilen-12,8-olide	1.728621917	0.290449979	down	0.266618069
3a,7a,12a-Trihydroxy-5b-cholestan-26-al	1.771971418	0.298311873	down	0.396994715
4-Methyl-2-pentenal	3.449529364	0.020036483	down	0.088060896
